# Deficiency of the aryl hydrocarbon receptor in kidney epithelial cells does not influence the development of atherosclerosis

**DOI:** 10.1038/s41598-026-63808-1

**Published:** 2026-07-29

**Authors:** Rosanna Huchzermeier, Caron Romel, Kathrin Abschlag, Manuel Rogg, Christoph Schell, Emiel P. C. van der Vorst

**Affiliations:** 1 Department of Internal Medicine I, Aachen-Maastricht Institute for Cardio-Renal Disease (AMICARE), Institute for Molecular Cardiovascular Research (IMCAR), University Hospital Aachen, RWTH Aachen University, Aachen, Germany; 2 Institute for Cardiovascular Prevention (IPEK), Ludwig-Maximilians-Universität, Munich, Germany; 3 Institute of Surgical Pathology, Faculty of Medicine, Medical Center - University of Freiburg, Freiburg, Germany

**Keywords:** Aryl hydrocarbon receptor, Kidney epithelial cells, Atherosclerosis, Cardiology, Diseases, Medical research, Nephrology, Physiology

## Abstract

The aryl hydrocarbon receptor (AHR) is recognised as an important mediator of inflammatory processes, including those in kidney health. Patients suffering from kidney diseases have an increased risk of cardiovascular diseases, such as atherosclerosis. Since the AHR can be activated in the kidney by, e.g., tryptophan metabolites and contribute to inflammatory processes, we hypothesized that a deficiency of the *Ahr* in the kidney epithelium in mice fed a high-fat diet could impact the development of atherosclerosis by altering renal function. We analysed *Apoe*^*−/−*^*Cdh16*^*Cre*^*Ahr*^*fl/fl*^ mice in comparison to *Apoe*^*−/−*^*Ahr*^*fl/fl*^ after feeding a 12-week high-fat diet in terms of systemic inflammation, plaque, and kidney phenotype. Accordingly, we found that the absence of *Ahr* in the kidney epithelium did not affect atherosclerotic outcomes and, surprisingly, did not alter the kidney phenotype with respect to inflammation or fibrosis. We therefore concluded that a high-fat diet does not sufficiently drive an AHR-mediated response in kidney epithelium, which could contribute to plaque development.

**Supplementary Information:**

The online version contains supplementary material available at 10.1038/s41598-026-63808-1.

## Introduction

Cardiovascular diseases (CVDs) are among the leading causes of death worldwide. With an increase of 93% CVD cases from 1990 to 2019, the death rate also rose by 53.72%^1^. Despite the high number of incidents, treatment options remain limited. Therefore, identifying new therapeutic targets is imperative.

One of the main causes of CVD is atherosclerosis, a chronic inflammatory condition characterised by plaque formation in arterial walls^2^. Due to endothelial dysfunction, which is provoked by several risk factors such as hyperlipidaemia, hypertension, smoking, or diabetes^3^, circulating lipoproteins invade the vessel wall, where they undergo oxidation^4^. In this reaction, a cascade of downstream inflammatory events is activated. Monocytes are recruited to the activated endothelium and migrate into the intima, where they differentiate and, through lipid uptake, become foam cells. During disease progression, smooth muscle cells are activated and form a fibrotic cap, while the plaque core, due to macrophage death and insufficient efferocytosis, becomes necrotic^5^. The formation of this fibrous cap primarily contributes to plaque stability. In contrast to stable plaques, vulnerable plaques pose the risk of rupture and, consequently, the risk of thrombus formation with major clinical events such as stroke or myocardial infarction^6^.

The kidney is a major modulator of cardiovascular disease. Both the heart and kidney are linked through a complex interplay of physiological and pathological processes^7^. Disorders characterised by acute or chronic dysfunction in either the heart or the kidneys that lead to functional impairment of the other organ are referred to as cardiorenal syndromes^7^. Several parameters concerning normal kidney function can majorly impact cardiac health. For instance, inflammatory pathways in the kidney play an important role in cardiovascular outcomes^8^.

One important immuno-inflammatory regulator is the aryl hydrocarbon receptor (AHR), which is highly expressed in the kidney. The aryl hydrocarbon receptor is a ligand-activated transcription factor whose activation by environmental toxins and endogenous metabolites is associated with oxidative stress, inflammation, and endothelial dysfunction^9^. Altering the expression of various genes, including phase I detoxification genes such as cytochrome P450 (CYP) monooxygenases (Cyp1a1 or Cyp1a2), induces various physiological and toxicological effects^10^. Beyond the originally discovered functions in detoxification, more recent studies have shed light on AHR’s role in inflammation^11^. By regulating the transcription of diverse genes relevant to inflammatory responses, such as interleukin 10 (IL-10), interleukin 17 (IL-17), and interleukin 22 (IL-22) or interacting with other inflammatory regulators like nuclear factor kappa-light-chain-enhancer of activated B cells (NF-κB), the AHR is a considerable target in many inflammatory diseases^12^.

Several recent studies suggest that the AHR plays a role in kidney health. On the one hand, patients with chronic kidney disease (CKD) exhibited signs of elevated AHR activation, as evidenced by increased CYP1A1 levels, which also seemed to correlate with increased cardiovascular risk^13^. Besides CKD, AHR activity is also correlated with other renal pathologies, like diabetic nephropathy and renal cell carcinoma. In these pathologies, AHR activation is correlated with increased inflammation, oxidative stress, and fibrosis, highlighting its impact on kidney health^14^.

The notion that AHR is an important kidney modulator associated with increased cardiovascular risk, combined with its role in inflammation and immunomodulation, raises the question of whether AHR in the kidney also affects cardiovascular outcomes. In a previous study, we demonstrated that global *Ahr* deficiency has a major impact on atherosclerotic plaque development^15^, affecting inflammatory mechanisms and lipid metabolism, although the specific cell types involved remained elusive. Based on this, we hypothesized that mice lacking the *Ahr* in kidney epithelial cells would alter their inflammatory and fibrotic response and, consequently, exhibit altered atherosclerotic plaque formation after feeding a high-fat diet. To investigate this hypothesis, we created pro-atherogenic *Apoe*^*−/−*^ mice lacking the *Ahr* specifically in kidney epithelial cells, using a tamoxifen-induced *Cdh16*^*cre*^ model.

## Materials and methods

### Animals

*Apoe*^*−/−*^*Ahr*^*fl/fl*^ mice were generated by cross-breeding *Apoe*^*−/−*^ and *Ahr*^*fl/fl*^ mice. To induce a specific *Ahr*-deficiency in kidney epithelial cells, *Apoe*^*−/−*^*Ahr*^*fl/fl*^ were crossbred with *Apoe*^*−/−*^*Cdh16*^*Cre/ERT2*^ mice. Both male and female mice were used. Experiments were started at 8 weeks of age (weight-range 18–24 g). After injection of tamoxifen (50 mg/kg body weight, i.p., Sigma–Aldrich; Cat#T5648 in Miglyol, Caelo) for 5 consecutive days to induce the Cre-activity, the mice were fed a high-fat diet (containing 21.1% crude fat, 17.3% crude protein, 5.0% crude fibre, 4.2% crude ash, 14.4% starch, 34.3% sugar & 0.21% cholesterol, Sniff TD88137) starting at 8 weeks of age. After 12 weeks, the mice were euthanised with non-antagonizable anaesthesia (10 mg/kg xylazine and 100 mg/kg ketamine) with subsequent blood collection through retro-orbital puncture. After flushing the vasculature with 10 ml ice-cold PBS, the organs were harvested. All animal studies were approved by the Landesamt für Natur, Umwelt und Verbraucherschutz Nordrhein-Westfalen, Germany, approval number 81-02.04.2019.A363). All procedures are in accordance with the guidelines outlined in Directive 2010/63/EU of the European Parliament on the protection of animals used for scientific purposes.

### Flow cytometry

Blood was collected in EDTA-buffered collection tubes. Erythrocyte lysis buffer (150 mM NH4Cl; 10 mM KHCO3; 215 0.1 mM diNaEDTA) was applied for 20 min. The reaction was stopped by adding Hank’s buffer (HBSS, 0.06% 0.5 M 216 EDTA, 0.6% BSA, pH 7.4). The blood was incubated with the following antibody mix: CD45 (APC-Cy7, Invitrogen), CD115 (FITC, Invitrogen), GR1 (V500, Biolegend), CD11b (PE-Cy7, Invitrogen), B220 (eF450, Invitrogen), CD3 (PerCP, Invitrogen), CD4 (APC, eBioScience), CD8 (PE, eBioScience). The flow cytometric analysis was performed using the BD FACS Canto with the FACS Diva Software (BD Bioscience). The subsequent analysis was performed with FlowJo 10.0 (BD BioScience) using the following gating strategy: total leukocytes (CD45+), classical monocytes (CD45 + CD115+GR1+), non-classical monocytes (CD45 + CD115+GR1-), neutrophils (CD45+,GR1+), B cells (CD45 + CD115-GR1-B220+), T cells (CD45 + CD115-GR1-CD3+).

### Atherosclerosis lesion analysis

After fixation in 4% paraformaldehyde, hearts were embedded in Tissue-Tek O.C.T compound (Sakura) for transverse cryo-sectioning, aortic arches were embedded in paraffin. The size of atherosclerotic plaques was quantified using Hematoxylin-Eosin (H&E) staining in the aortic roots (H&E, Sigma) and arches. Collagen deposition in the aortic root plaques was determined using Masson’s trichrome staining. The macrophage infiltration in aortic root plaques was measured by applying immunofluorescence co-staining. After antigen-retrieval in citrate buffer and blocking of unspecific binding sites with PBS/0.01% bovine serum albumin/0.0125% horse serum, the Galectin 3 (Mac2, Cedarlane) antibody in PBS containing 0.015% of the blocking solution was applied overnight at 4 °C. The secondary antibody DL488 was incubated for 1 h at RT, followed by a Hoechst 33,342 (ThermoFisher Scientific)-staining to stain the nuclei. After mounting with Fluoromount-G (ThermoFisher Scientific), images were taken using a Leica DMLB microscope and a charge-coupled device camera. The images were analysed using QuPath^16^. For each staining, the average of three sections per mouse was taken.

### Plasma lipid analysis

Plasma cholesterol and triglyceride levels were measured using enzymatic assays (Roche diagnostics) according to the manufacturer’s protocol.

### Kidney lysates

10 mg of snap-frozen kidney were thawed on ice and lysed in M-PER™ mammalian protein extraction reagent (ThermoFisher Scientific), including 1% HALT™ Protease Inhibitor Cocktail (ThermoFisher Scientific) and 1% HALT™ Phosphatase Inhibitor Cocktail (ThermoFisher Scientific). The tissue was lysed by homogenisation with a metal bead at 50 Hz for 5 min using the TissueLyser LT (Qiagen) and subsequently sonicated for 5 min using a sonicator bath. Protein concentration was measured using the Nanodrop One Microvolume UV-VIS Spectrophotometer (ThermoFisher Scientific).

### ELISA

Inflammatory cytokine levels in kidney lysates were measured using commercially available ELISA kits from ThermoFisher Scientific. following the manufacturer’s protocol. The cytokine concentration was normalised to the total protein amount in the tissue lysates.

### Histological analysis of the Kidney

Histological analysis of kidney sections stained with periodic acid–Schiff (PAS) was performed as previously described^17^. A four-tiered semiquantitative histology score was applied to assess interstitial fibrosis (IF) and tubular atrophy (TA) (score of 0 indicates < 5% of tubules are affected, 1: 5%–25%, 2: >25%–50%, 3: >50%), tubular necrosis (0: negative, 1: <5%, 2: 5%–25%, 3: >25%), proteinaceous or cellular casts in tubules (0: negative, 1: <5%, 2: 5%–25%, 3: >25%). Five-tiered histology scores were applied to assess glomerular sclerosis (GS), peritubular inflammation (PTI) and tubular cysts (0: negative, 1: <5%, 2: 5%–25%, 3: >25%–50%, 4: >50%).

### RNA isolation and RT-qPCR

5-10 mg of snap-frozen kidney were homogenised in TRIzol (Qiagen) with a metal homogeniser for 5 min at 50 Hz at 4 °C. RNA was subsequently isolated using the Qiagen RNeasy Micro Kit according to the manufacturer’s protocol, including a genomic DNA digestion step. RNA concentration was measured using the Nanodrop One Microvolume UV-VIS Spectrophotometer (ThermoFisher Scientific). cDNA was synthesised using the M-MLV reverse transcriptase at 37 °C for 1 h. RT-qPCR was performed using the PowerUp™ SYBR ™ Green master Mix and the following primers: *mCcl2-Fw*: GCTGGAGAGCTACAAGAGGATCA, *mCcl2-Rv*: TCTCTCTTGAGCTTGGTGACAAAA, *mIl-6-Fw*: ATGGATGCTACCAAACTGGAT, *mIl-6-Rv*: TGAAGGACTCTGGCTTTGTCT, *mMmp-2-Fw*: CTTCACGCTCTTGAGACTTTGGTTC, *mMmp-2-Rv*: GATAACCTGGATGCCGTCGTG, *mMmp-9-Fw*: CCTGGAACTCACACGACATCTTC, *mMmp-9-Rv*: TGGAACTCACACGCCAGAA, *mTimp1-Fw*: TGGGTCCCCAGAAATCAACG, *mTimp1-Rv*: CAAGCAAAGTGACGGCTCTG, *mTnf-Fw*: CATCTTCTCAAAATTCGAGTGACAA, *mTnf-Rv*: TGGGAGTAGACAAGGTACAACCC, *mTiparp-Rv*: GAACCCCACCAAGTGTCTGTAA, *mAhrr-Fw*: AAGCCCATTCAGAAGCGGAGG, *mAhrr-Rv*: CCAGCTCTGTATTGAGGCGGT, *mCyp1a1-Fw*: TGAAGGTGGTAGTTCTTGGAGC, *mCyp1a1-Rv*: CATGATCTAGGTGGCTGCTTGG, *mβ-Act-Fw*: CAACGAGCGGTTCCGATG, *mβ-Act-Rv*: GCCACAGGATTCCATACCCAA, *mTiparp-Fw*: AGAAGCCAACTCTCGGGGTC,

Primer specificity was controlled by melt curve analysis. The relative mRNA expression was calculated using the 2^ΔΔCt method, with β-Actin as the reference gene.

### Statistics

Statistical analysis was conducted using GraphPad Prism 10 (GraphPad Software Inc). The Shapiro-Wilk test was used to test for a Gaussian normal distribution. Statistical outliers were identified using the ROUT test. Depending on the data distribution, comparisons between two groups were made using either the unpaired two-tailed Student’s t-test with Welch’s correction or the Mann-Whitney U test, as appropriate. A two-tailed *P* value < 0.05 was considered statistically significant. Data are represented as mean ± standard error of mean (SEM). *P < 0.05.

## Results

### Kidney epithelial cell *Ahr* deficiency results in leukocytosis in females

**Fig. 1:**
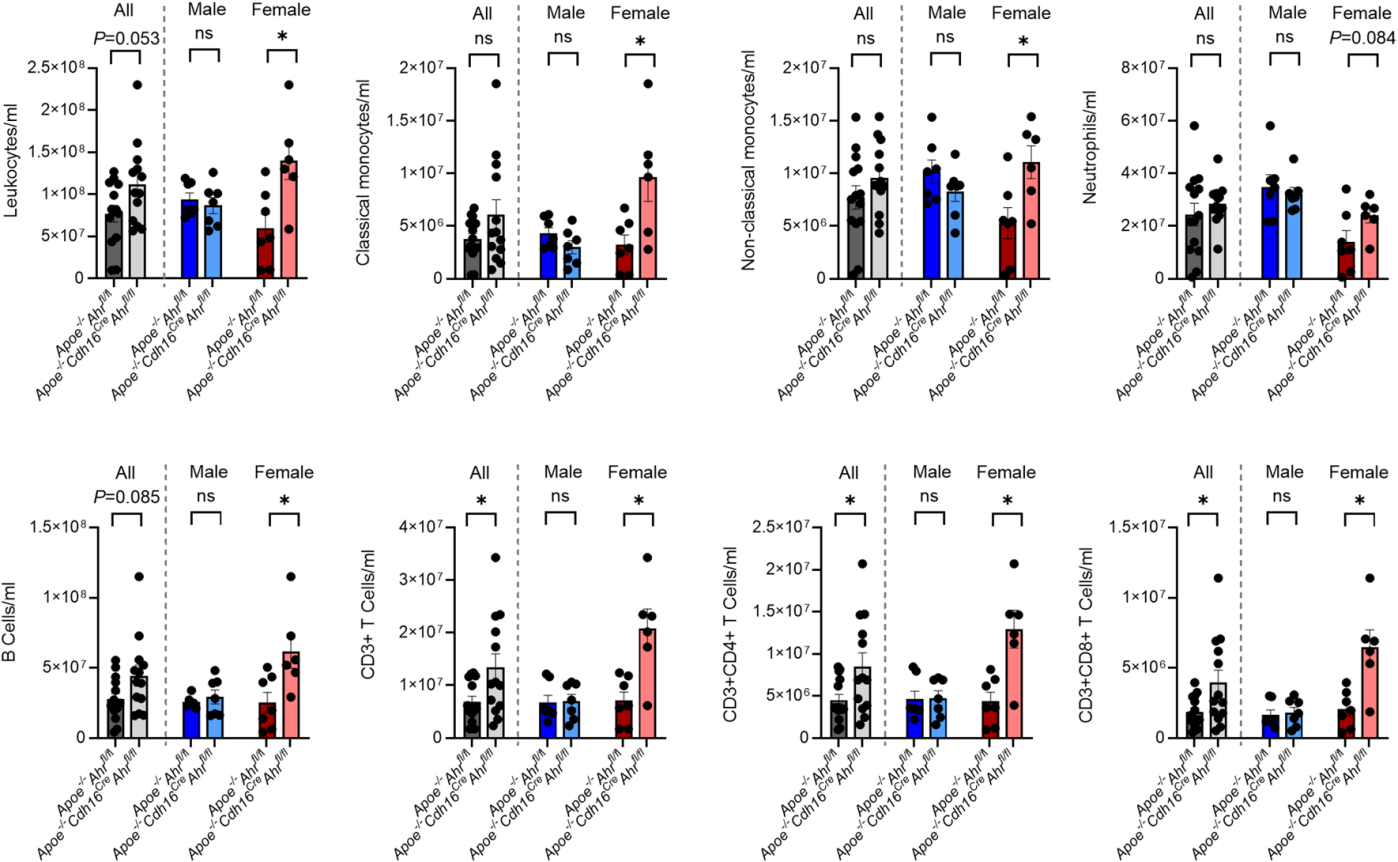
Female *Apoe*^*−/−*^*Cdh16*^*cre*^*Ahr*^*fl/fl*^mice show increased circulating leukocyte levels compared to *Apoe*^*−/−*^*Ahr*^*fl/fl*^mice after a 12w HFD. All results are obtained from *Apoe*^*−/−*^*Cdh16*^*cre*^*Ahr*^*fl/fl*^ and *Apoe*^*−/−*^*Ahr*^*fl/fl*^ mice after 12 weeks of HFD, with combined sexes, and separated by males and females. Blood total leukocyte and leukocyte subset counts are shown. Combined *n* = 13–14, male *n* = 7, female *n* = 6–7, graphs represent mean ± SEM.

To investigate whether an *Ahr deficiency* in kidney epithelial cells impacts the development of atherosclerosis, we crossbred *Apoe*^*−/−*^*Ahr*^*fl/fl*^ with *Apoe*^*−/−*^*Cdh16*^*Cre*^ mice. Confirming the successful *Ahr-*deletion, we could identify significantly reduced mRNA expression of key AHR target genes cytochrome P450 (*Cyp1a1*), Ahr-repressor (*Ahrr*), and poly-[ADP-ribose]-polymerase 7 (*Tiparp*) in kidney tissue of *Apoe*^*−/−*^*Cdh16*^*Cre*^ mice in comparison to *Apoe*^*−/−*^*Ahr*^*fl/fl*^ mice (**Supplementary Figure ****S1**). After a 12-week HFD, we first investigated whether circulating leukocytes were altered, as HFD-induced intestinal barrier disruption can lead to leukocytosis. Interestingly, the absence of kidney epithelial cell *Ahr* resulted in leukocytosis only in female mice, whereas leukocytes in male mice were unaffected. This leukocytosis in female mice was observed across all leukocyte subsets, including both myeloid and lymphoid cells (Fig. 1).

### Atherosclerotic plaque development is not affected by a kidney epithelial cell *Ahr* deficiency

**Fig. 2:**
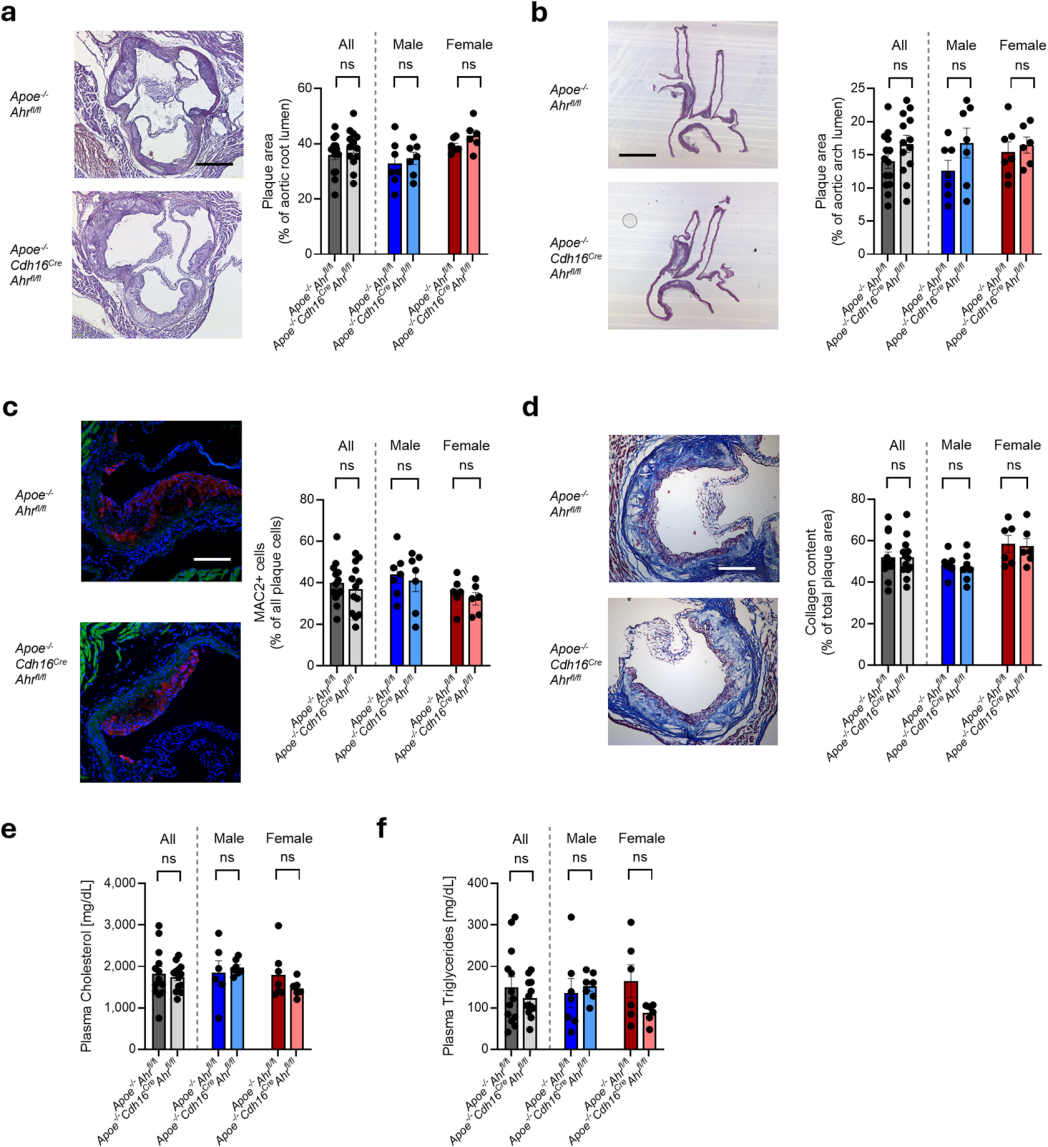
Kidney epithelial *Ahr *deficiency does not affect atherosclerosis development. All results are obtained from *Apoe*^*−/−*^*Cdh16*^*cre*^*Ahr*^*fl/fl*^ and *Apoe*^*−/−*^*Ahr*^*fl/fl*^ mice after 12 weeks of HFD, with combined sexes, and separated by males and females. **(a)** Representative pictures of HE-stained aortic roots (scale = 200 μm) with quantification of plaque lesion size. **(b)** Representative pictures and quantification of plaque lesion size in aortic arches (scale = 2 mm). **(c)** Representative pictures and quantification of Mac2^+^ cells in aortic root plaques. (**d)** Representative pictures and quantification of collagen content in aortic root plaques. **(e)** Plasma cholesterol levels. **(f)** Plasma triglyceride levels. Combined *n* = 13–14, male *n* = 6–7, female *n* = 6–7, graphs represent mean ± SEM.

Based on the increased circulating leukocytes, we expected that female mice in particular would also exhibit increased atherosclerotic plaque development. However, the plaque size was not altered in the aortic roots or arches (Fig. 2A-B). Consistent with unaltered leukocyte counts in male mice, we observed no differences in plaque size in male mice lacking *Ahr* in kidney epithelial cells compared with male controls. In addition to plaque size, we did not observe any differences in plaque phenotype, as lesional macrophage accumulation and plaque collagen content in aortic roots were unaltered in both male and female mice (Fig. 2C-D). Additionally, as a hallmark of atherosclerosis, we measured plasma cholesterol and triglyceride levels. Both remained unaffected in male and female mice (Fig. 2E-F).

### Kidney epithelial cell *Ahr* deficiency does not affect renal inflammation or fibrosis markers

**Fig. 3:**
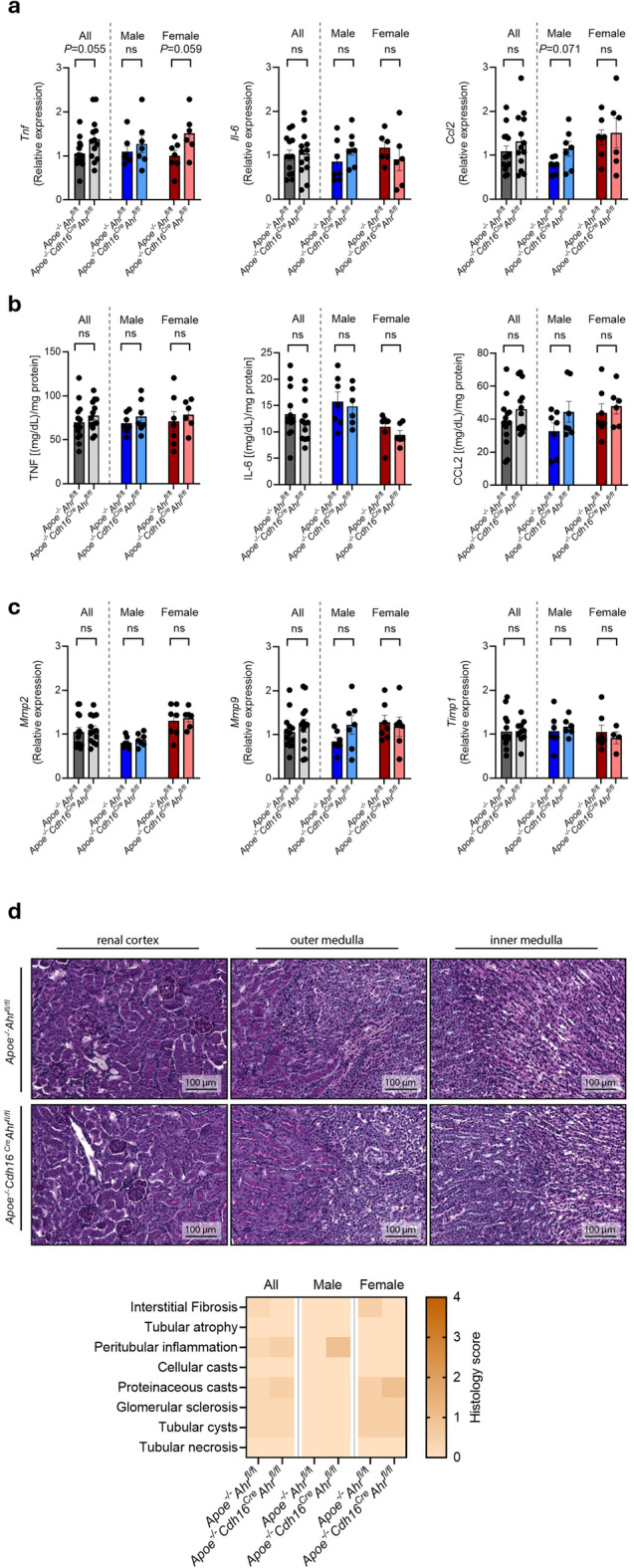
The kidney phenotype is unaffected by the lack of kidney epithelial *Ahr*. All results are obtained from *Apoe*^*−/−*^*Cdh16*^*cre*^*Ahr*^*fl/fl*^ and *Apoe*^*−/−*^*Ahr*^*fl/fl*^ mice after 12 weeks of HFD, with combined sexes, and separated by males and females. **(a)** Relative mRNA expression of *Tnf*,* Il6*, and *Ccl2* in kidney tissue. **(b)** Protein cytokine levels of TNF, IL-6, and CCL2 in kidney lysates. **(c)** Relative mRNA expression of *Mmp2*,* Mmp9*, and *Timp1* in kidney tissue. (**d**) Representative pictures of PAS-stained kidney sections and histological scoring (combined *n* = 4, male *n* = 2, female *n* = 2). Combined *n* = 10–14, male *n* = 6–7, female *n* = 4–7, unless stated otherwise. Graphs represent mean ± SEM.

Due to unexpected, unaltered plaque phenotypes in the mice, we aimed to determine whether the absence of *Ahr* in kidney epithelial cells affects the kidney phenotype. On a genetic level, the mRNA expression of the inflammatory cytokines tumour necrosis factor (*Tnf*), interleukin 6 (*Il6*), and CC-chemokine ligand 2 (*Ccl2*) was not significantly different; however, *Tnf* expression in female *Apoe*^*−/−*^*Cdh16*^*cre*^*Ahr*^*fl/fl*^ mice and the *Ccl2* expression in male *Apoe*^*−/−*^*Cdh16*^*cre*^*Ahr*^*fl/fl*^ mice tended to be increased in comparison to their sex-matched controls (Fig. 3A). However, at the protein level, none of the inflammatory cytokines was significantly altered (Fig. 3B). Similarly, the expression of the fibrotic markers matrix metalloproteinase 2 (*Mmp2*), matrix metalloproteinase 9 (*Mmp9*), and tissue inhibitor of metalloproteinase 1 (*Timp1*) was unaffected (Fig. 3C). To further identify potential histological changes within the kidneys, we performed a pathological scoring based on PAS-stained kidney sections. However, we did not observe any significant differences in kidney phenotypes among genotypes and sexes (Fig. 3D).

Taken together, these results suggest that the genetic deficiency of *Ahr* in kidney epithelial cells does not affect the development of atherosclerotic plaques, systemic inflammation, or lipid levels. Moreover, surprisingly, the kidney itself does not show major alterations in inflammation and fibrosis.

## Discussion

The heart and kidney are functionally linked through a complex interplay of physiological and pathological processes^7^. AHR has been identified as an important kidney modulator associated with increased cardiovascular risk. Based on our previous work demonstrating that a global *Ahr* deficiency has a major impact on atherosclerosis plaque development, together with the notion that AHR is highly expressed in kidney epithelial cells^18,19^, we hypothesized that a deficiency of the *Ahr* in the kidney epithelial cells of atherosclerotic mice could ameliorate the disease progression of atherosclerosis and thereby at least partly explain the observations from the global *Ahr* deficient model^15^. However, contrary to our expectations, we observed no effects on plaque development or the kidney phenotype, suggesting that other cell types are responsible for the observed effects on lipid metabolism and atherosclerosis.

These results are rather surprising, since it has been shown previously that the AHR primarily contributes to kidney health and cardiovascular-kidney crosstalk^20^. For instance, it has been shown that an *Ahr* deficiency in renal tubular epithelial cells in CKD mice led to decreased renal fibrosis and senescence^21^. In the context of CKD, the *Ahr* has been recognized as an important mediator. The activation of the AHR by uremic toxins has been shown to lead to increased kidney inflammation and disease progression^22^. In idiopathic membranous nephropathy, inflammation and oxidative stress are regulated via activation of the AHR-mediated NF-κB pathway, highlighting AHR’s importance in kidney inflammation^23^. In contrast, in the context of acute kidney injury, AHR activation was shown to inhibit the expression of pro-inflammatory cytokines^21^. Since the AHR is generally linked to inflammation in various organs and even specific cell types^24^, we expected changes in the kidney’s inflammatory status upon *Ahr* deficiency, which did not seem to be the case herein. Additionally, despite the known fact that the AHR can contribute to kidney fibrosis^25^, we did not observe major effects in the expression of fibrotic markers in the kidneys lacking the *Ahr*.

Several studies have shown that the AHR is a highly cell-type- and context-dependent actor^26^. Therefore, it seems likely that, in our mouse model, the diet did not sufficiently provoke an AHR-mediated inflammatory response. Interestingly, we observed that in female mice, blood leukocyte levels were higher when *Ahr* was absent in kidney epithelial cells. A similar leukocytosis was already observed in *Apoe*^*−/−*^ mice with a global *Ahr*-deficiency. While leukocytosis also did not appear to have a major impact on plaque development, the whole-body *Ahr* deficiency had detrimental effects on lipid metabolism and liver inflammation, whereas the kidney-specific deletion showed none of these effects. As the mice in the study herein also showed no consequences regarding plaque development, it is unclear what impact this leukocytosis has and which downstream events trigger it.

Patients suffering from CKD show a tremendously increased risk for cardiovascular events^27^, which highlights the importance of kidney health towards cardiac health. Patients with CKD also show increased levels of uremic toxins, such as indoxyl sulphate and kynurenine in their blood^27^. A change in diet can alter the gut microbiome^28^. As a consequence, an altered microbiome also affects the production of microbial metabolites, including several tryptophan metabolites^29^. Since the AHR is a major receptor for tryptophan, we suggest that, upon CKD development, the role of the AHR might be more impactful in the development of atherosclerosis and therefore warrants further study in this context.

Recent studies, combined with our findings, suggest that the AHR in kidney epithelium plays a versatile yet conflicting role. Our data indicate that, under an HFD, renal AHR does not significantly affect the kidney phenotype or atherosclerotic plaque development.

## Supplementary Information

Below is the link to the electronic supplementary material.

Supplementary Material 1

## Data Availability

Raw data are available upon reasonable request.
