# Supplementary material for: Deficiency of the aryl hydrocarbon receptor in kidney epithelial cells does not influence the development of atherosclerosis
Source: Sci Rep. 2026 Jul 29;16:23537. doi: 10.1038/s41598-026-63808-1 (PMC13416111; doi:10.1038/s41598-026-63808-1)
Supplement: Supplementary file 1 — Supplementary Material 1 [file 41598_2026_63808_MOESM1_ESM.pdf]

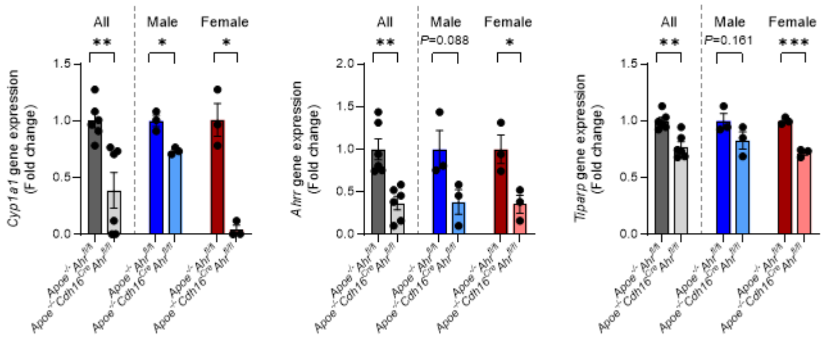

Supplementary Figure S1. Reduced AHR signaling in mice lacking kidney epithelial *Ahr*.

X-fold mRNA expression of AHR target genes *Cyp1a1, Ahrr,* and *Tiparp* in kidney lysates from representative $Apoe^{-/-}Cdh16^{cre}Ahr^{fl/fl}$ and $Apoe^{-/-}Ahr^{fl/fl}$ mice after a 12 weeks of HFD. Combined n=6, male n=3, female n=3, graphs represent mean ± SEM.
